# Identifying high-yield low-emission pathways for the cereal production in South Asia

**DOI:** 10.1007/s11027-017-9752-1

**Published:** 2017-07-22

**Authors:** Tek B. Sapkota, Jeetendra P. Aryal, Arun Khatri-Chhetri, Paresh B. Shirsath, Ponraj Arumugam, Clare M. Stirling

**Affiliations:** 1 International Maize and Wheat Improvement Centre (CIMMYT), NASC complex, New Delhi, 110012 India; 2 International Maize and Wheat Improvement Centre (CIMMYT), Texcoco, Mexico; 3 CGIAR Research Program on Climate Change, Agriculture and Food Security (CCAFS), Borlaug Institute for South Asia (BISA)/CIMMYT, NASC Complex, New Delhi, 110012 India

**Keywords:** Greenhouse gas emissions, Climate change, Cereal systems, High-yield low-emission pathway

## Abstract

Increasing agricultural production to meet the growing demand for food whilst reducing agricultural greenhouse gas (GHG) emissions is the major challenge under the changing climate. To develop long-term policies that address these challenges, strategies are needed to identify high-yield low-emission pathways for particular agricultural production systems. In this paper, we used bio-physical and socio-economic models to analyze the impact of different management practices on crop yield and emissions in two contrasting agricultural production systems of the Indo-Gangetic Plain (IGP) of India. The result revealed the importance of considering both management and socio-economic factors in the development of high-yield low-emission pathways for cereal production systems. Nitrogen use rate and frequency of application, tillage and residue management and manure application significantly affected GHG emissions from the cereal systems. In addition, various socio-economic factors such as gender, level of education, training on climate change adaptation and mitigation and access to information significantly influenced the adoption of technologies contributing to high-yield low-emission pathways. We discussed the policy implications of these findings in the context of food security and climate change.

**Electronic supplementary material:**

The online version of this article (doi:10.1007/s11027-017-9752-1) contains supplementary material, which is available to authorized users.

## Introduction

Global agricultural greenhouse gas (GHG) emissions are increasing. In the past 50 years, emissions from agriculture, forestry and fisheries have nearly doubled due to an increase in global agricultural production (Smith et al. 2014). This is driven largely by an increase in demand for food and changes in food consumption patterns, particularly in developing countries. Recent analysis suggests that trends in population growth and food demand will result in a further 30% increase in global GHG emissions from agricultural by 2050 with Asian and African countries accounting for most of the increase (Tubiello et al. 2014).

Synthetic fertilizer is one of the major and fastest growing emission sources in agriculture (Camargo et al. 2013; Shcherbak et al. 2014), together with paddy rice (*Oryza sativa* L.) cultivation and burning of crop residues (Wassmann et al. 2000; INCCA 2010; Boateng et al. 2017). Energy and fertilizer consumption is particularly high in intensive cereal production systems. For example fertilizer and chemical energy (e.g. pesticides, herbicides etc) inputs comprise about 45% of the total energy consumed for production of rice, wheat (*Triticum aestivum* L.) and maize (*Zea mays* L.) (Khan et al. 2009), about 60% of which is due to nitrogen fertilizer alone. On the other hand, projections indicate that production of food crops such as rice, wheat and maize needs to increase over the coming decades to meet food demand (Jat et al. 2011). Given the necessity to increase crop production and limited scope for horizontal expansion, fertilizer consumption is expected to increase, thereby augmenting emissions from agriculture. Therefore, production systems guided by key concerns of sustainability are necessary in order to increase food production without compromising environmental integrity.

Many developing countries identified agriculture and allied sectors as one of the priority areas for emission reduction in their Intended Nationally Determined Contributions (INDCs) to the United National Framework Convention on Climate Change (UNFCCC) (Richards et al. 2016). Similarly, climate change adaptation and mitigation-related policies and programs in many developing countries highlight the need for sustainable increase in agricultural production and a reduction of emissions where possible. These countries possess immense mitigation potential for land use in agriculture and have targeted this sector to reduce their carbon footprints (CCAFS 2015). However, current understanding of the effect of various management options on GHG emissions from crops and the enabling socio-economic factors that influence their adoption is limited. This dearth of information constrains the development of evidence-based strategies and targets for low-carbon agricultural development.

Reductions in GHG emissions from agricultural sector can be achieved through improved agronomic practices and adoption of precision input management and enhanced resource use efficiencies (Godfray et al. 2010; Sapkota et al. 2015a, b; Verge et al. 2007). Yield improvement and emission reduction are also directly influenced by farmers’ decisions to implement farm technologies and/or practices. Farming decisions are influenced by farm size, income level, land ownership, access to market and credits and other socio-economic variables (Keil et al. 2015; Khatri-Chhetri et al. 2016; Mottaleb et al. 2016). However, there remains a major knowledge gap in terms of the relationship between agricultural emissions and socio-economic conditions which needs to be better understood in order to develop appropriate emission reduction strategy for agriculture.

The overall aim of this paper is to identify high-yield low-emission development pathways in cereal production systems. To achieve this, specific objectives are as follows: (i) to identify various technologies and farm management practices that influence GHG emissions and (ii) to explore household socio-economic factors that determine the adoption of low-emission technologies and management practices at the farm level. By identifying the important determinants of GHG emissions, this paper explores a possible pathway to produce or maintain higher yields with lower GHG emissions in two major, but different, production systems in the Indian Indo-Gangetic Plains (IGP) through the use of a GHG emission tool, household survey and statistical models. The study has important global implications because the IGP is the major food bowl of India producing food for about 40% of India’s 1.2 billion population (Saharawat et al. 2010). India is the third largest GHG emitter in the world (Ge et al. 2014) with agriculture being the second largest source of GHG emission accounting for 18% of the gross national emissions (INCCA 2010). The country has recently declared a voluntary goal of reducing the emission intensity of its GDP by 33–35% and placed emphasis on land-based mitigation measures (India’s INDC to UNFCCC, http://www.moef.nic.in/climate-change-docs-and-publications). As the third largest emitter in the world, such a commitment has the potential for substantial impacts on global emissions. The state of Haryana in India represents a high-input production system with relatively high productivity whereas Bihar represents a low-input production system with correspondingly low productivity. By using these two states of India as exemplary study, we discuss the policy implications of the findings in the context of food security and climate change.

## Materials and methods

### Study area and data collection

**Fig. 1:**
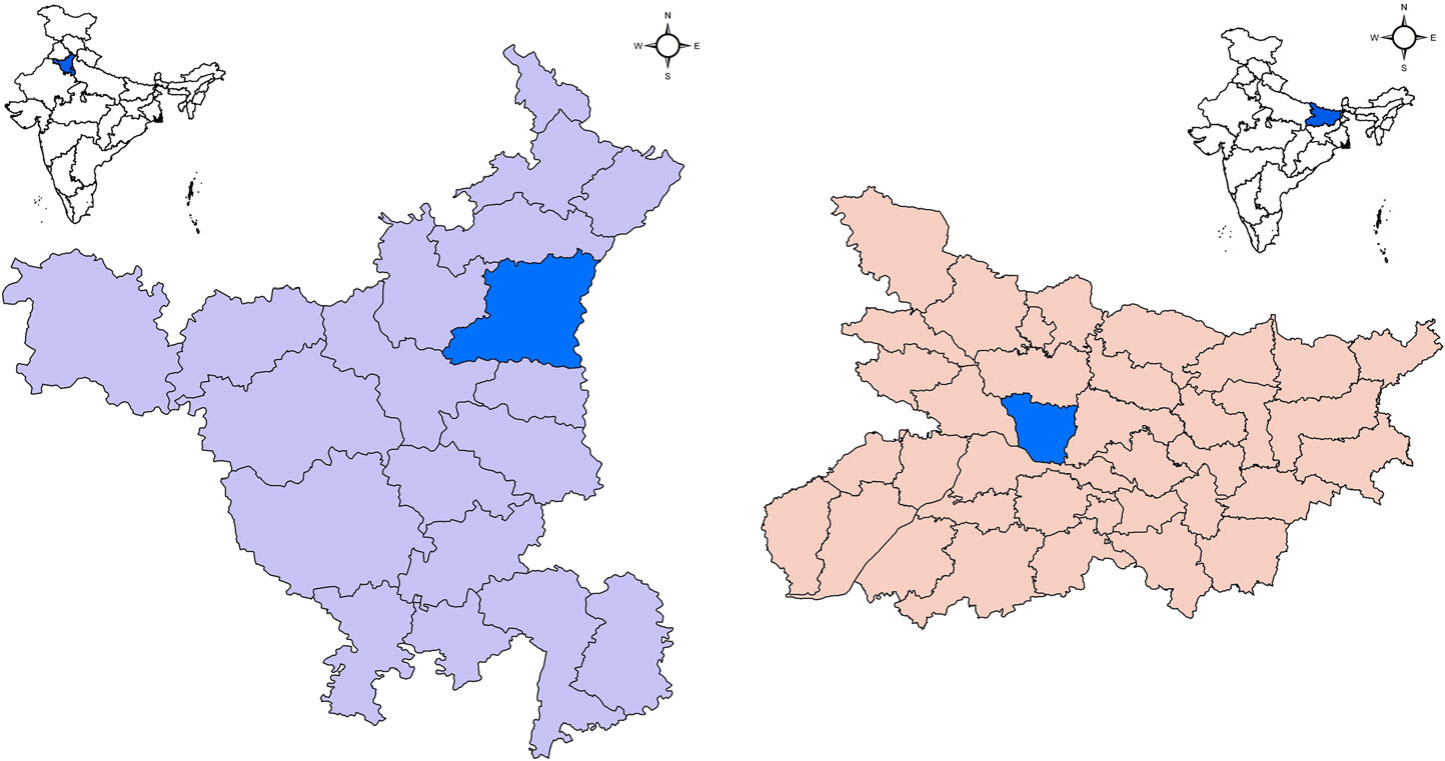
Location of Karnal and Vaishali districts within Haryana and Bihar States of India

Data for this study were derived from a household survey conducted in two districts: Karnal district of Haryana state and Vaishali district of Bihar state in India (Fig. 1) in 2013. Karnal and Vaishali represent high and low input production systems typical of the western and eastern IGP, respectively. Cereal production in the IGP is GHG intensive compared to other regions in South Asia. Annual GHG emissions resulting from the production of rice, wheat and maize in the Indian IGP are 113,388, 20,727 and 1632 Gg CO_2_eq, respectively (calculated using the average emissions reported in Vetter et al. 2017).

An overview of the agro-ecological conditions of the study sites are given in Table 1. The household survey comprised 626 and 641 randomly selected households in Karnal and Vaishali, respectively. In this survey, farmers were interviewed to obtain information on crop production, socio-economic and demographic conditions, climate risks in agriculture and adaptation and mitigation measures. Within household, farmers manage multiple plots for different crops (rice, wheat, maize) under different management conditions. From each plot, information regarding tillage operations and fuel use, crop establishment, agronomy (nutrient, water, weed and pest), yield and residue management were obtained. Total applied N in the plots was calculated as the sum of the N from di-ammonium-phosphate (DAP), urea, farm-yard-manure (FYM) and crop residue (where applicable) and assuming an N content of FYM as 0.5% (Tandon 1994) and that of crop residues as 0.8% (Dobermann and Fairhurst 2002). The soil data needed for the model but not collected during the survey were obtained from The Global Soil Dataset for Earth System Modeling (Shangguan et al. 2014).

**Table 1:** Basic agro-ecological characteristics of the study sites

Particulars	Karnal	Vaishali
Latitude	29.6857° N	25.6838° N
Longitude	76.9905° E	85.3550° E
Crops included in the study	Rice and wheat	Rice, wheat and maize
Average annual rainfall (mm)	669	1168
Agro-ecology	Warm arid and semiarid subtropics	Warm sub-humid subtropics
Soil texture	Medium	Medium
Soil pH	6.4–8.2	7
Soil organic matter (%)	0.41–0.72	0.72
Soil bulk density (Mg m^−3^)	1.5	1.5
Soil CEC (cmol_c_ kg^−1^)	20.122–22.867	20.122
Rice area (ha)	171,517	57,633
Wheat area (ha)	171,412	48,136
Maize area (ha)	287	33,939

### Estimation of GHG emission and global warming potential

We used the CCAFS Mitigation Options Tool (CCAFS-MOT) to estimate GHG emissions (Feliciano et al. 2016) which allows assessment of GHG emissions as a function of management practice and enables the user to examine and optimize different management options. CCAFS-MOT combines several empirical models to estimate GHG emissions from different land uses. The tool recognizes context-specific factors that influence GHG emissions such as pedo-climatic characteristics, production inputs and other management practices at the field as well as the farm level. The model allows to evaluate the performance of the production system from a GHG emission perspective, both in terms of land-use efficiency and efficiency per unit of product. The model calculates background and fertilizer-induced emissions based on multivariate empirical model of Bouwman et al. (2002) for nitrous oxide (N_2_O) and nitric oxide (NO) emissions and the model of FAO/IFA (2001) for ammonia (NH_3_) emission. Emissions from crop residues returned to the field were calculated using IPCC N_2_O Tier 1 emission factors. Similarly, emissions from the production and transportation of fertilizer were based on Ecoinvent database (Ecoinvent Center 2007). Changes in soil C due to tillage were based on Powlson et al. (2016). Similarly, effect of manure and residue management on soil C were based on IPCC methodology as in Ogle et al. (2005) and Smith et al. (1997). Emissions of CO_2_ from soil resulting from urea application or liming were estimated using IPCC methodology (IPCC 2006). To estimate the total GHG emissions from the production systems, i.e. global warming potential (GWP), all GHGs were converted into CO_2_-equivalents (CO_2_e) using the GWP (over 100 years) of 34 and 298 for CH_4_ and N_2_O, respectively (IPCC 2013). Yield-scaled GWP of each crop was determined by dividing the total GWP by grain yield.

### Identifying determinants of GHG emissions

We used the multiple regression model in Stata 13.1 (Cameron and Trivedi 2009) to estimate the impact of different inputs and management factors on GHG emissions. Total emissions from crop production are affected by the rate and application frequency (one-time application or multi-split application) of nitrogen fertilizer, tillage practice (e.g. conventional or zero-tillage), application of manure and incorporation of crop residues. The dependent variable is the emission of individual GHGs and total GWP (all in CO_2_e ha^−1^) from rice, wheat and maize production. The empirical model used to identify determinants of the emission is as follows:

1 $$ {E}_i=\alpha +\beta {X}_n+\delta {X}_m+\gamma {X}_t+{\in}_i $$

where *E*_*i*_ denotes the emission of individual GHGs and total GWP for the ith plot, *X*_*n*_ represents the total nitrogen application (kg/ha) on different crops, *X*_*m*_ is the matrix of management practices, *X*_*t*_ refers to the tillage method and ε is the usual error term. α, β, δ and γ are the parameters estimated.

After determining the effect of various management factors on the individual and total emissions, we explored the various farm and farmers’ characteristics which determine the choice of management strategies, for example, nitrogen fertilizer, manure and retention of crop residues that influence GHG emission. We broadly categorized all nitrogen sources as organic and inorganic nitrogen. The empirical model used is as follows:

2 $$ {Y}_i=\alpha +\beta {X}_s+\delta {X}_r+\gamma {X}_k+\rho {X}_d+{\in}_i $$

where *Y*_*i*_ denotes the use of nitrogen in different forms, *X*_*s*_ is the matrix of household socio-economic characteristics, *X*_*r*_ is the matrix of household access to productive resources, *X*_*k*_ is the matrix of knowledge enhancing activities such as training and access to information services, *X*_*d*_ is represents the matrix of crop and location-related variables. α, β, δ, γ and *ρ* are the parameters estimated. We also used similar empirical model to estimate the effect of various household characteristics on yield and yield-scaled GWP (kg CO2e Mg^−1^ grain yield of rice, wheat and maize).

Changes in emissions of individual GHGs as well as total GWP with N rate were illustrated by regressing emissions with N rate using ‘ggplot2’ package (Wickham 2009) in ‘R’ software (R Core Team 2016). To identify the GHG efficient production system and corresponding N rate, relationships were established between yield and yield-scaled GWP under different N application ranges, as described by Bellarby et al. (2014). For this, the total N applied was binned into 20 kg N ha^−1^ range, i.e. 0–20, 20–40, ……… 220–240 kg N ha^−1^, and corresponding yield and yield-scaled GWP were plotted together. Finally, a stylized framework (Cui et al. 2014) of grain yield and GHG emission was developed to evaluate different GHG-efficient production pathways.

## Results

### Grain yield

**Fig. 2:**
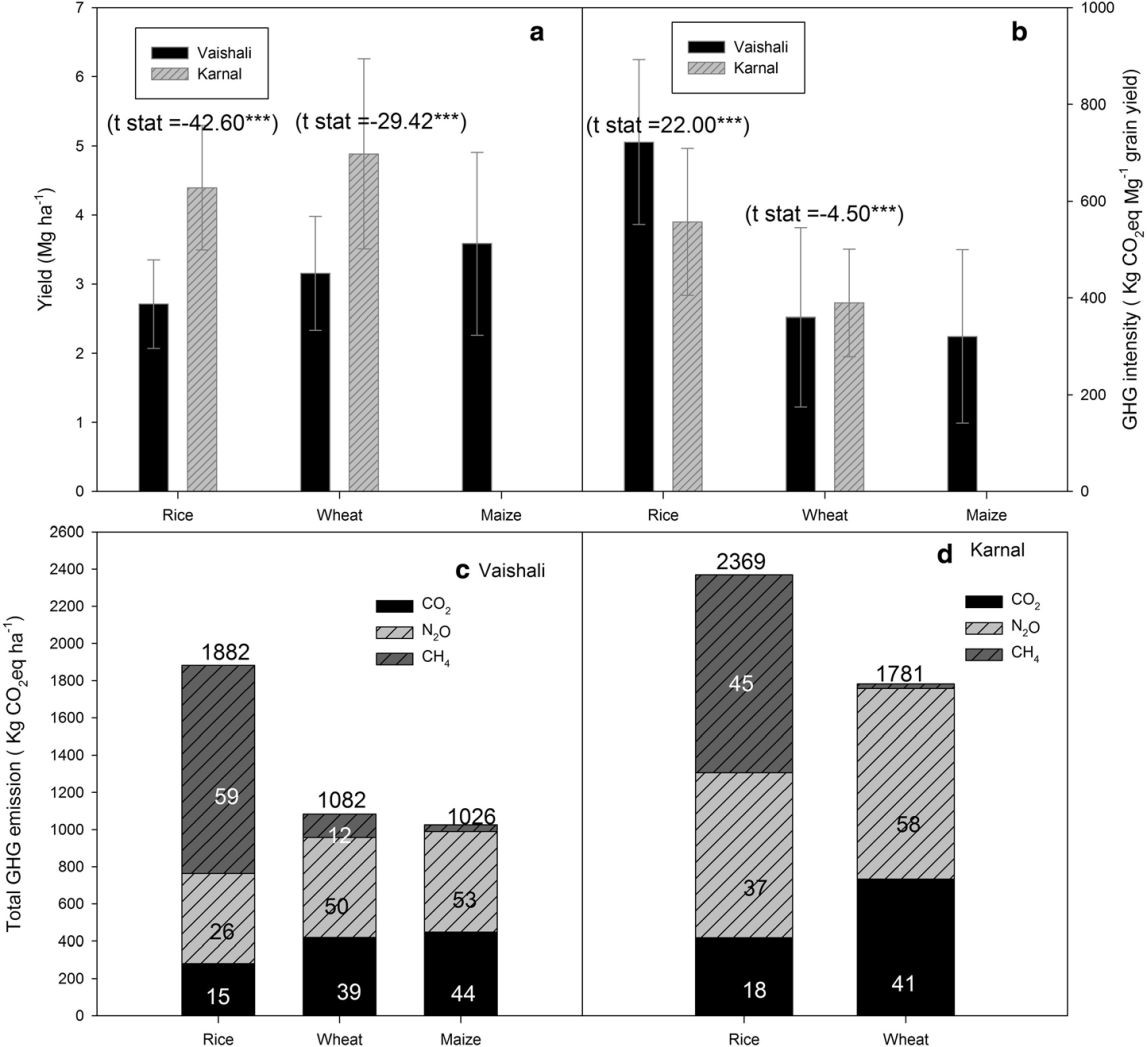
*Upper panel*: grain yield (**a**) and yield-scaled GWP (**b**) of rice, wheat and maize in Karnal and Vaishali. *t* statistic and corresponding level of significance indicate the mean difference between Karnal and Vaishali. *Lower panel*: total GWP for rice, wheat and maize in Vaishali (**c**) and Karnal (**d**) showing the contribution of various GHGs. In a *bar*, the number within each stack indicates the percentage contribution of individual gas to total GWP

On average, rice and wheat grain yields were, respectively, 162 and 150% greater in Karnal than Vaishali (Fig. 2a). Maize grain yield was 3.4 ± 0.1 Mg ha^−1^ in Vaishali, none of the respondents in Karnal grew maize. Yield-scaled GWP (i.e. emission intensity) was much higher in rice (722 ± 170 kg CO_2_e Mg^−1^ in Vaishali and 557 ± 151 kg CO_2_e Mg^−1^ in Karnal) than in wheat (359 ± 185 kg CO_2_e Mg^−1^ in Vaishali and 389 ± 111 kg CO_2_e Mg^−1^ in Karnal) and maize (320 ± 179 kg CO_2_e Mg^−1^) (Fig. 2b).

### GHG emissions and GWP

Crop emissions were significantly greater in Karnal than Vaishali (compare Fig. 2c, d). Of all three crops, rice had the largest GWP with CH_4_ being the major contributing GHG (Fig. 2c, d). By contrast, N_2_O and CO_2_ contributed to the major portion of GWP in wheat and maize accounting for between 50 to 58% and 39 to 44% of the total, respectively, in both regions.

### Agronomic management and GHG emission

Table 2 presents the effect of various management factors on emission of individual gases and total GWP in rice, wheat and maize. The result shows that the total amount of N applied (from inorganic and organic sources) affected all the GHGs, resulting in a significant effect on total GWP in all three crops. Adoption of zero tillage in rice and wheat decreased CH_4_ and CO_2_ emission but did not affect N_2_O emissions. This resulted into a significant reduction in total GWP. Higher numbers of N split increased both CO_2_ and N_2_O emissions resulting in significantly higher total GWP in all three crops (Table 2). Substituting a portion of the N supply through farm yard manure (FYM) and crop residue generally reduced the emissions of CO_2_, had no effect on N_2_O but increased CH_4_ emission thereby significantly increasing total GWP in rice. *R* ^2^ values at the bottom of the Table 2 indicate that included factors in the model explained 70% of the variability in total CH_4_ emission in rice and over 95% of the variability in CO_2_, N_2_O and total GWP in all three crops. As four out of five variables included in the model are related to fertilizer input, it is evident that fertilizer input (N in particular) is an important driver of overall GHG emission from rice, wheat and maize.

**Table 2:** Effect of various management factors on the emission of individual greenhouse gases and total global warming potential (GWP; all in CO_2_e ha^−1^) from rice, wheat and maize production

Management factors	Rice				Wheat			Maize		
	CH4	CO2	N2O	Total GWP	CO2	N2O	Total GWP	CO2	N2O	Total GWP
Total nitrogen	−0.90***	3.92***	4.73***	7.75***	3.66***	4.77***	8.42***	3.86***	4.46***	8.31***
	(0.12)	(0.05)	(0.04)	(0.13)	(0.04)	(0.04)	(0.08)	(0.10)	(0.11)	(0.21)
Zero tillage	−574.55***	−584.38***	−1.21	−1160.15***	−228.81***	4.73	−224.08***			
	(10.02)	(22.61)	(4.00)	(18.80)	(7.60)	(4.40)	(10.83)			
N split	12.87***	51.64***	67.59***	132.10***	51.01***	61.66***	112.66***	52.32***	81.66***	133.98***
	(4.84)	(4.16)	(4.70)	(9.19)	(4.13)	(5.28)	(9.31)	(8.96)	(11.35)	(20.17)
Manure	9.31***	−3.30***	0.77***	6.77***	−3.61***	0.00	11.58***	−3.71***	0.48	11.95***
	(0.68)	(0.08)	(0.07)	(0.67)	(0.06)	(0.08)	(0.14)	(0.30)	(0.40)	(0.70)
Crop residue	18.43***	−16.03***	−0.10	2.31*	−5.82***	0.49	−5.33***	−5.36*	−1.19	−6.55
	(1.21)	(0.53)	(0.31)	(1.21)	(0.33)	(0.30)	(0.59)	(3.07)	(4.29)	(7.32)
Constant	1118.71***	−149.18***	−93.35***	876.19***	−23.25***	−84.47***	−107.72***	−36.18**	−92.75***	−128.93***
	(6.96)	(8.85)	(9.73)	(17.63)	(8.37)	(10.86)	(18.99)	(15.21)	(20.15)	(35.16)
*R* ^2^	0.70	0.94	0.97	0.95	0.97	0.98	0.98	0.94	0.94	0.96
No. of observation	2120	2120	2120	2120	2075	2075	2075	369	369	369

Note: *, ** and *** refer to 10, 5 and 1% level of significance, respectively. Standard errors are reported in parentheses

### GHG emission in relation to N input

Irrespective of the type of crop, total N input from organic and inorganic sources had a significant positive effect on N_2_O, CH4 and total GWP (Fig. S1). Averaged over the crops and locations, 1.15% of applied N was emitted directly as N_2_O.

**Fig. 3:**
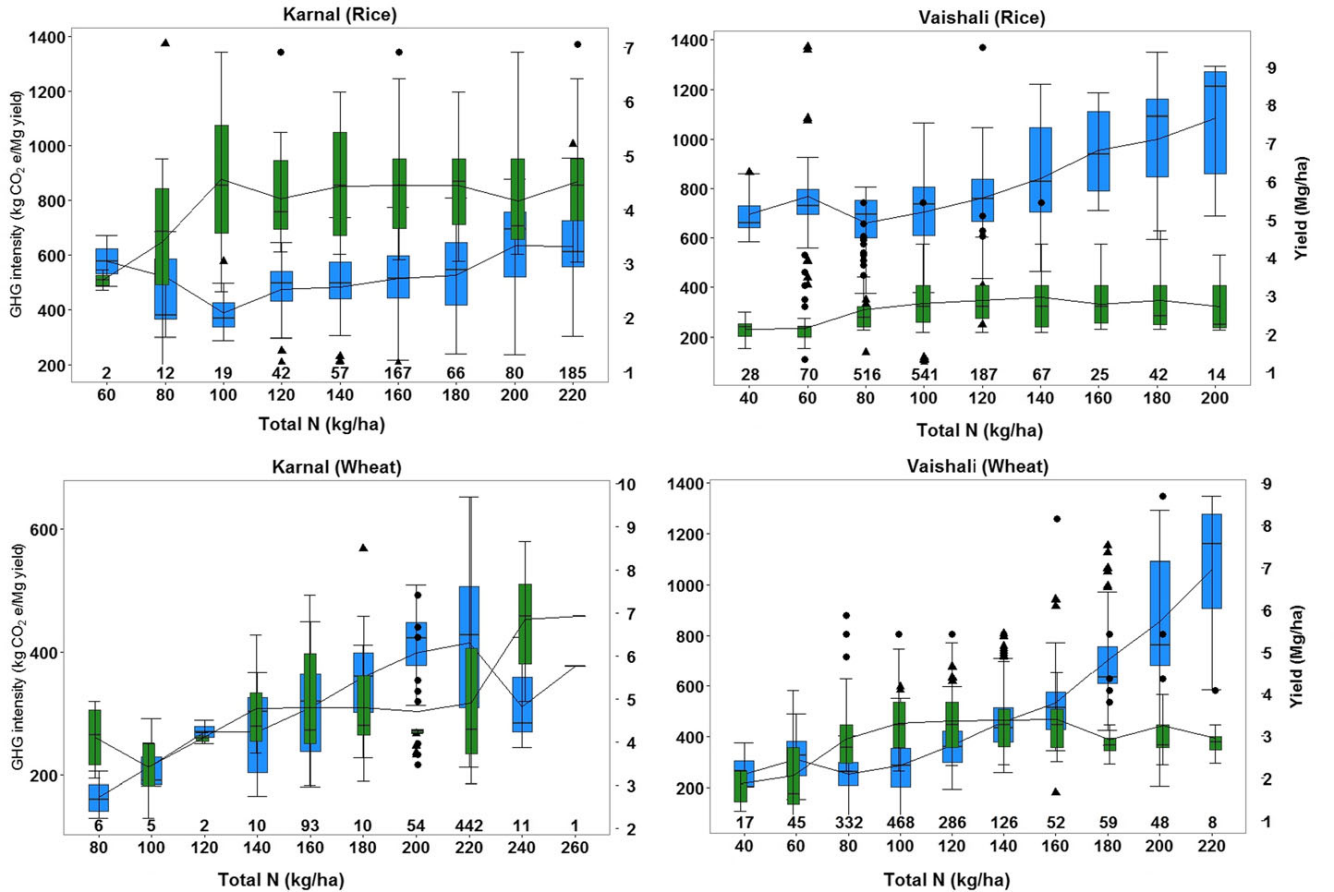
Box plot of yield-scaled GWP (*blue*) and corresponding observed grain yield (*green*) for each bin of N rate in Vaishaili and Karnal. Each N bin is 20 kg N application range represented by the maximum value of the range (e.g. N bin 40, 60,… represents N application range between 20–40, 40–60, … and so on). The number at the bottom to each *bar* represents the number of samples in each N bin

Figure 3 shows the response of total grain yield and GHG emission intensity (kg CO_2_eq Mg^−1^ grain yield) to applied N in rice and wheat. The majority of the farmers in Karnal applied more than 100 kg of N ha^−1^ whereas those in Vaishali applied less than 100 kg N ha^−1^. In general, grain yield of rice was higher in Karnal than in Vaishali for all N bins. In rice, GHG emission intensity was higher in Vaishali than in Karnal across all N bins. Irrespective of the sites, rice yield leveled off with increased N application rate beyond 100 kg ha^−1^ whilst GHG emission intensity continued to increase with N rate. Wheat yield, on the other hand, increased up to 100 kg N ha^−1^ in Vaishali and at a higher rate of about 140 kg N ha^−1^ in Karnal. With the exception of few wheat farmers in Karnal, N application rates above 100 kg ha^−1^ in Vaishali and 140 kg N ha^−1^ in Karnal did not enhance the grain yield but significantly increased the GHG emission intensity. Wheat farmers in Vaishali who applied above 140 kg N ha^−1^ got no marginal yield gain as a result of additional fertilizer thus resulting into higher GHG emission intensities.

### Factors affecting N input, yield and GHG emission

Table 3 presents the effect of various household characteristics on the yield of rice, wheat and maize. Rice and wheat yields were significantly higher in Karnal than in Vaishali, and yield of all crops was higher in male-headed household and significantly so for rice and maize. The effect of the household head’s educational level was observed for rice yield whereas that of the spouse was observed for wheat yield. The effect of household size was negative and significant for wheat and maize yield. Remittance has a small but significant effect on wheat and maize yield. Similarly, size of landholding had significantly positive effect on maize yield. Ownership of a thresher had a significantly positive effect on rice and wheat yield whereas tractor ownership had a significantly negative effect on maize yield. Climate change-related training and access to seed from local agro-vets had significantly positive effect on wheat and maize yield. Of the different methods of receiving information related to agriculture, use of information and communication technology (for example, use of mobile phones to receive agricultural information including weather forecasts and application of farm inputs) was found to have a significant positive impact on yield of all three crops. Interestingly, access to credit and information from neighboring farmers were a few of the criteria that had a consistent and negative effect on yield across all three crops.

**Table 3:** Effect of various household characteristics on the yield (kg ha^−1^) of rice, wheat and maize

Household characteristics	Rice	Wheat	Maize
General caste HH	18.67	66.61	−112.50
	(36.58)	(52.00)	(170.65)
Male-headed HH	168.14**	82.87	604.11*
	(66.93)	(97.40)	(327.87)
Literate HH head	120.46***	78.23	−88.93
	(38.12)	(53.94)	(164.85)
Literate spouse of HH head	−8.35	140.59**	−48.00
	(39.08)	(55.26)	(170.72)
Family size	2.87	−17.74**	−49.09*
	(6.10)	(8.67)	(27.16)
Remittance received (in INRs)	0.00	0.00**	0.01***
	(0.00)	(0.00)	(0.00)
Livestock owned (TLU)	−9.79	5.72	−91.55
	(11.24)	(15.90)	(92.82)
Cultivated land (in ha)	−8.78	0.92	498.04***
	(6.07)	(8.22)	(137.46)
Credit access	−60.04*	−226.63***	−608.15***
	(34.07)	(48.58)	(142.84)
Tractor owned	−86.81	141.35	−1135.66**
	(67.29)	(95.17)	(574.95)
Thresher owned	572.23***	747.63***	1110.55
	(157.48)	(221.00)	(903.21)
Training on climate change	60.80	476.39*	2418.61*
	(201.67)	(283.40)	(1391.42)
Training on soil and water management	266.06	−105.25	−1616.14
	(184.91)	(268.34)	(1219.35)
Training on seed management	82.35	164.79	.
	(83.87)	(117.63)	.
Training on crop rotations	−127.14***	110.11	155.43
	(48.74)	(68.80)	(166.36)
Information obtained from extension service	86.94	−295.83**	−181.78
	(106.04)	(146.87)	(510.70)
Information obtained from neighboring farmers	−81.06**	−231.64***	−236.34
	(40.67)	(57.80)	(157.86)
Information obtained from private companies	−50.06	106.37*	348.84**
	(42.14)	(59.90)	(154.86)
Information and communication technology	165.68*	352.25**	567.87**
	(95.50)	(142.45)	(244.39)
Haryana state (base category: Bihar)	1648.72***	1527.69***	
	(54.81)	(77.07)	
Constant	2571.00***	3220.68***	3544.40***
	(74.71)	(109.20)	(373.07)
*R* ^2^	0.55	0.41	0.23
No. of Observation	2058	2016	351

Note: *, ** and *** refer to 10, 5 and 1% level of significance, respectively. Standard errors are reported in parentheses

*HH* household, *INR* Indian National Rupees, *TLU* total livestock unit

Overall, the amount of organic as well as inorganic fertilizer N input was significantly different in the study sites (Table 4). In general, fertilizer N input was significantly higher in Karnal than in Vaishali and lower in rice and maize compared with wheat. Organic, inorganic and total N inputs were positively and significantly related to total livestock units in the household implying that those households with more livestock tend to apply more N fertilizer. As in the case of yield, access to credit had a significantly negative effect on inorganic and thus total N input but no effect on organic N. Training on crop rotation had a significant positive effect on organic N input and negative effect on inorganic N.

**Table 4:** Effect of various household characteristics on organic, inorganic and total N input (kg ha^−1^) averaged over rice, wheat and maize

	Total N	Inorganic N	Organic N
Family size	0.13	0.25	−0.12
	(0.36)	(0.34)	(0.11)
Livestock owned (TLU)	1.28***	0.81*	0.46***
	(0.48)	(0.46)	(0.16)
Cultivated land (ha)	0.10	0.19	−0.09**
	(0.29)	(0.29)	(0.05)
Credit access	−3.46*	−3.54*	0.08
	(2.04)	(2.00)	(0.74)
Tractor owned	3.63	2.68	0.95
	(4.35)	(4.42)	(0.74)
Thresher owned	1.05	2.43	−1.37
	(8.43)	(8.20)	(1.93)
Participated in training on climate change	11.92	11.65	0.27
	(8.30)	(8.33)	(1.17)
Participated in training on soil and water	1.00	4.14	−3.14***
	(6.06)	(5.88)	(1.06)
Participated in training on seed management	−4.03	−5.38	1.35*
	(3.38)	(3.33)	(0.78)
Participated in training on crop rotation	−2.96	−7.01**	4.04***
	(2.84)	(2.72)	(1.14)
Information obtained from extension service	−8.16*	−6.37	−1.79**
	(4.89)	(4.91)	(0.72)
Information obtained from neighbor	4.90*	7.01***	−2.11**
	(2.63)	(2.59)	(0.86)
Information obtained from private company	−3.21	−4.96**	1.75
	(2.26)	(2.17)	(1.14)
Information and communication technology	1.10	3.93	−2.82***
	(4.36)	(4.48)	(0.86)
Literate HH head	0.91	2.15	−1.24
	(2.07)	(1.99)	(0.87)
Literate spouse of HH head	1.15	2.01	−0.86
	(2.13)	(2.10)	(0.57)
Rice (base category: wheat)	−16.06***	−9.60***	−6.45***
	(1.16)	(0.90)	(0.77)
Maize (base category: wheat)	−4.96**	−0.80	−4.17***
	(2.33)	(2.15)	(1.06)
Karnal (base category: Vaishali)	80.10***	76.61***	3.49***
	(2.59)	(2.51)	(0.96)
Constant	100.32***	89.59***	10.72***
	(3.26)	(2.96)	(1.74)
*R* ^2^	0.62	0.63	0.08
No. of observation	4544	4544	4544

Note: *, ** and *** refer to 10, 5 and 1% level of significance, respectively. Standard errors are reported in parentheses

*HH* household, *TLU* total livestock unit

Yield-scaled GWP of rice and wheat was significantly lower in cases where the household head was literate as compared to the ones where household heads were illiterate (Table 5). In wheat, yield-scaled GWP was also higher in the households with more livestock. Maize yield-scaled GWP was higher in the households with smaller landholdings. Similarly, yield-scaled GWP of wheat and maize was significantly lower in the household owning a thresher, who participated in various agricultural trainings.

**Table 5:** Effect of various household characteristics on yield-scaled GWP (kg CO2e Mg^−1^ grain yield of rice, wheat and maize

	Rice	Wheat	Maize
Literate HH head	−23.26**	−26.04*	−7.92
	(11.83)	(14.63)	(28.87)
Literate spouse of HH head	5.02	−22.68*	35.61
	(13.42)	(11.66)	(40.43)
Family size	−0.12	−1.03	4.38
	(2.14)	(2.11)	(3.44)
Livestock owned (TLU)	−0.07	8.67***	13.50
	(3.18)	(2.84)	(15.43)
Cultivated land (ha)	−0.99	−1.16	−48.62**
	(1.72)	(1.04)	(24.03)
Credit access	−1.09	9.38	20.99
	(11.59)	(12.72)	(32.49)
Tractor owned	6.49	7.18	221.87***
	(21.31)	(18.41)	(50.47)
Thresher owned	−62.60	−107.76***	−248.98***
	(41.36)	(32.38)	(62.91)
Participated in trainings			
Climate change	−79.86	−34.32	−60.63
	(50.26)	(32.57)	(81.97)
Soil and water management	−26.30	−11.29	5.60
	(34.55)	(31.38)	(44.68)
Seed management	−67.50***	−8.23	.
	(20.95)	(18.25)	.
Crop rotation	12.06	13.53	17.21
	(17.91)	(19.15)	(25.24)
Agricultural information received			
Extension service	19.07	−21.69	−21.70
	(22.27)	(19.29)	(59.46)
Neighbors and relatives	48.64***	15.25	−13.02
	(16.02)	(16.46)	(40.90)
Seed company	−5.59	−5.96	−51.85**
	(14.95)	(18.95)	(24.28)
ICT	87.41**	22.91	22.30
	(36.15)	(27.29)	(32.48)
Haryana state (base category: Bihar)	−126.13***	21.88	
	(16.46)	(16.94)	
Constant	710.02***	372.48***	307.76***
	(19.83)	(24.17)	(44.05)
*R* ^2^	0.21	0.04	0.07
No. of observation	2112	2066	366

Note: *, ** and *** refer to 10, 5 and 1% level of significance, respectively. Standard errors are reported in parentheses

*HH* household, *TLU* total livestock unit, *ICT* Information Communication Technology

A multivariate logit model was used to examine the likelihood of adoption of yield-enhancing and emissions-reducing technologies at the plot level. Many household and plot level characteristics had significant influence on the adoption of these technologies (Table 6). For example, the household with general caste, high level of education, large land holding, access to information/agro-advisory and those who received training on climate change were likely to adopt zero tillage. Similarly, those who received training on climate change, soil and water management and seed management and those having access to agricultural credit tend to adopt split application of nitrogen. Farmers having large holding size and those who received training and crop rotation were more likely to apply manure in their field. Results also show that farmers practicing intensive, input-based agriculture in Karnal are adopting zero tillage and split dose nitrogen use more than the farmers in Bihar. Recently, many state and local agricultural development-related programs, CGIAR research centers and other organizations are promoting these technologies in Haryana so that the adoption rate is gradually increasing over the time.

**Table 6:** Factors explaining the likelihood to adopt zero tillage, split application of nitrogen and manure application

	Zero tillage	N split	Manure
State dummy (Karnal = 1; Vaishali = 0)	0.91***	2.12***	0.08
	(0.13)	(0.10)	(0.07)
Caste dummy (general caste = 1; otherwise = 0)	0.36***	0.47***	−0.03
	(0.11)	(0.05)	(0.05)
HH head sex dummy (male = 1; female = 0)	−0.71**	−0.30***	0.13***
	(0.35)	(0.09)	(0.04)
Literate HH head	0.62***	−0.02	0.05
	(0.13)	(0.05)	(0.05)
Literate spouse of HH head	0.07	0.16***	−0.03
	(0.10)	(0.06)	(0.06)
Main occupation of HH head (agriculture = 1; otherwise = 0)	0.03	0.05	0.00
	(0.16)	(0.09)	(0.09)
Cultivated land (in ha)	0.05***	−0.04**	0.02***
	(0.01)	(0.02)	(0.01)
Training on climate change	1.49***	0.43***	0.10
	(0.18)	(0.16)	(0.13)
Training on soil and water management	0.80**	1.00**	0.07
	(0.34)	(0.46)	(0.26)
Training on seed management	−0.15	0.55***	−0.06
	(0.38)	(0.18)	(0.24)
Train on crop rotation	−0.65***	0.01	0.36***
	(0.25)	(0.06)	(0.06)
Information obtained from neighboring farmers	−0.01	0.11**	−0.08
	(0.13)	(0.05)	(0.06)
Information obtained from extension service	−0.38	0.10	−0.16
	(0.24)	(0.16)	(0.15)
Information obtained from private companies	−0.13	−0.03	0.01
	(0.19)	(0.05)	(0.06)
ICT	0.17***	−0.18	−0.04
	(.06)	(0.11)	(0.13)
Credit access	0.19**	0.35***	−0.09**
	(0.09)	(0.05)	(0.04)
Constant	−2.66***	0.45***	−1.07***
	(0.33)	(0.15)	(0.15)
Pseudo *R* ^2^	0.58	0.35	0.22
No. of observation	4544	4544	4544

*HH* household, *ICT* Information Communication Technology

## Discussion

### Grain yield and global warming potential of rice, wheat and maize

Average grain yields in our study sites were slightly higher than those reported in the Directorate of Economics and Statistics of the Government of India (http://eands.dacnet.nic.in) for respective crops in the respective states. Average grain yields observed in our study were, however, less than those reported from on-station trials in Bihar (Jat et al. 2014) and also those reported from the on-farm nutrient management trials of wheat in Haryana (Sapkota et al. 2014).

The total GWP for rice production in our study was within the range (1027–2632 kg CO_2_e ha^−1^) reported from India (Bhatia et al. 2005; Malla et al. 2005; Datta et al. 2009) but smaller than the values reported by Linquist et al. (2012) (3900 kg CO_2_e ha^−1^) through global meta-analysis. Relatively higher emissions reported in Linquist’s meta-analysis are probably because a large proportion of the data (77% of data points for rice) were from South-East Asia where rice is grown mostly under continuously flooded conditions leading to high CH_4_ emission. In our study sites, rice is mostly grown as a rainfed crop in Vaishali or intermittently irrigated crop in Karnal. Thus, the magnitude of CH_4_ emission from rice in our study (18–65 kg CH_4_ ha^−1^) was much smaller than those reported from South-East Asia and China (Zou et al. 2005; Ma et al. 2009; Zhang et al. 2010; Shang et al. 2011). The estimated total GHG emission from wheat and maize in our study was similar to those reported by Linquist et al. (2012) (1107–1238 kg CO_2_e ha^−1^) through meta-analysis of field measurements globally.

Higher total GHG emission from rice than wheat and maize (Fig. 2) was mainly driven by higher CH_4_ emission. This is because anaerobic conditions in rice field leads to methanogenesis (biochemical decomposition of organic matter in anaerobic environments), which is responsible for CH_4_ emission. Further, rice plants are important conduits of CH_4_ from soil to atmosphere sometime accounting for up to 90% of the total CH_4_ emission (Butterbach-bahl et al. 1997). The contribution of CH_4_ to the total GHG emission was nominal in the case of wheat and maize. In both rice and wheat, total GHG emissions were higher in Karnal than in Vaishali (Fig. 2) mainly driven by higher rates of N in Karnal than in Vaishali. GWP of wheat and maize was mainly driven by N_2_O and CO_2_ emissions related to fertilizer input. In this study, CO_2_ emissions refer to the balance of CO_2_ emissions/sequestration due to tillage and organic matter application (compost, manure and crop residues); CO_2_ emissions due to production and transportation of fertilizer; and CO_2_ emissions due to application of urea in the field.

Linquist et al. (2012) suggest that yield-scaled GWP is an appropriate integrated metric that addresses the dual goals of environmental protection and food security. In our study, yield-scaled GWP was almost two-fold greater in rice than wheat or maize (Fig. 2b). Evidently, higher yield-scaled GWP in rice than in wheat and maize was mainly due to CH_4_ emission from rice. Yield-scaled GWP in rice, in our study, was smaller than the value reported by Pathak et al. (2010) (1221 kg CO_2_e Mg^−1^) and larger than the value reported by Malla et al. (2005) (~210 kg CO_2_e Mg^−1^) for India but similar to the one reported by Linquist et al. (2012) (655 kg CO_2_e Mg^−1^). The GWP estimates of Pathak et al. (2010) are unrealistically high whilst the lower GWP estimates reported by Malla et al. (2005) were because the authors considered only CH_4_ and N_2_O emission in GWP calculation. Our estimates also include CO_2_ emission due to production, transportation and field application of fertilizer besides field emission of CH_4_ and N_2_O.

Yield-scaled GWP of wheat and maize production were slightly higher than the GWP value reported by Linquist et al. (2012) and by Malla et al. (2005) from the rice-wheat system of India. Again, this difference is primarily due to inclusion of CO_2_ emission arising from production, transportation and field application of fertilizer in our GWP calculation whereas GWP from other studies were solely based on field emission of CH_4_ and N_2_O.

### Agronomic, management and household characteristics contributing to reduce GHG emission

Our study indicates that both N_2_O and CH_4_ emissions are higher with higher N input (Fig. S1) leading to higher total GHG emission in all crops. Higher N input associated with number of livestock units (Table 3) indicates that farmers with more livestock in the household tend to apply more organic manure in addition to the usual amount of inorganic N. Lower yield-scaled emissions associated with literate household heads, larger holding size and thresher ownership (Table 5) suggest that richer and educated farmers intensify farming with better input-use-efficiency thereby reducing emissions while maintaining yield. Our findings are similar to those of Ju et al. (2016) from China, who also reported that large farms are more sensitive to fertilizer use thereby increasing production and reducing emission. Similarly, lower yield-scaled GWP in maize among the farmers receiving information from private seed companies suggest the higher yield from these farmers resulting from high yielding and quality seeds. Therefore, use of high yielding varieties (e.g. hybrids) to increase yield is one way forward to reduce emission intensity. Lower emission intensity with the farmers receiving training on climate change, seed and cropping system management (Table 5) shows that increasing farmers’ awareness and capacity in the area of agriculture and climate change is an important mechanisms to reduce GHG emission from agriculture. The negative relationship between credit access and N input (Table 3) and thus crop yield (Table 2) indicates that farmers in the study area are probably using agricultural credit for non-agricultural purposes, as has also been reported by Aryal et al. (2014). Higher GHG emissions with higher number of N-splits (Table 2) in this case were because farmers who apply fertilizer more frequently tend to apply more N fertilizer in total. Also, N_2_O emissions in our model are based on Bouwman et al. (2002) and do not take into account the effects of timing of N application. Although our results show no effect of substituting inorganic N with organic sources, it should be noted that N from the organic sources will not be fully available to crops in the same season and may induce N_2_O emissions in subsequent years also. Overall, these production technologies associated with precision nutrient management, tillage and residue management etc. also contribute to climate change adaptation and so could be promoted through appropriate programs and policies for adaptation-led mitigation in agriculture.

### Identifying high-yield low-emission pathways of cereal production

**Fig. 4:**
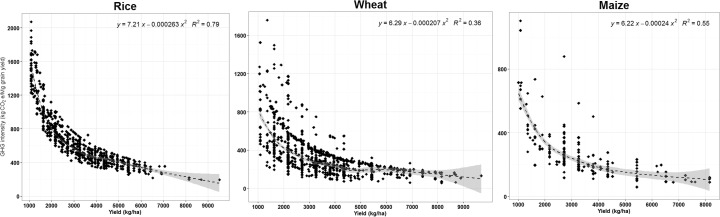
Relationship between yield-scaled GWP (kg CO_2_-eq Mg^−1^ grain yield) and grain yield of rice, wheat and maize

The results from both high-input (Karnal) and low-input (Vaishali) production systems clearly illustrate the dependence of GHG emissions on fertilizer usage, particularly N fertilizer (Table 2; Fig. 3). However, GHG emissions cannot be reduced only by reducing N fertilizer input due to trade-offs between yield and food security. Lower crop yields may also induce emissions elsewhere by bringing additional land into cultivation in order to meet the shortfall in food production (Bellarby et al. 2014). As adequate nutrient input is essential to increase and maintain crop production (Tittonell and Giller 2013), a valid approach under smallholder systems is to compute and compare emissions on a per-tonne-product basis, i.e. yield-scaled GWP or emission intensity. The strong negative correlation between grain yield and emission intensity for all three crops (Fig. 4) indicates that emission intensity can be reduced by increasing yield.

**Fig. 5:**
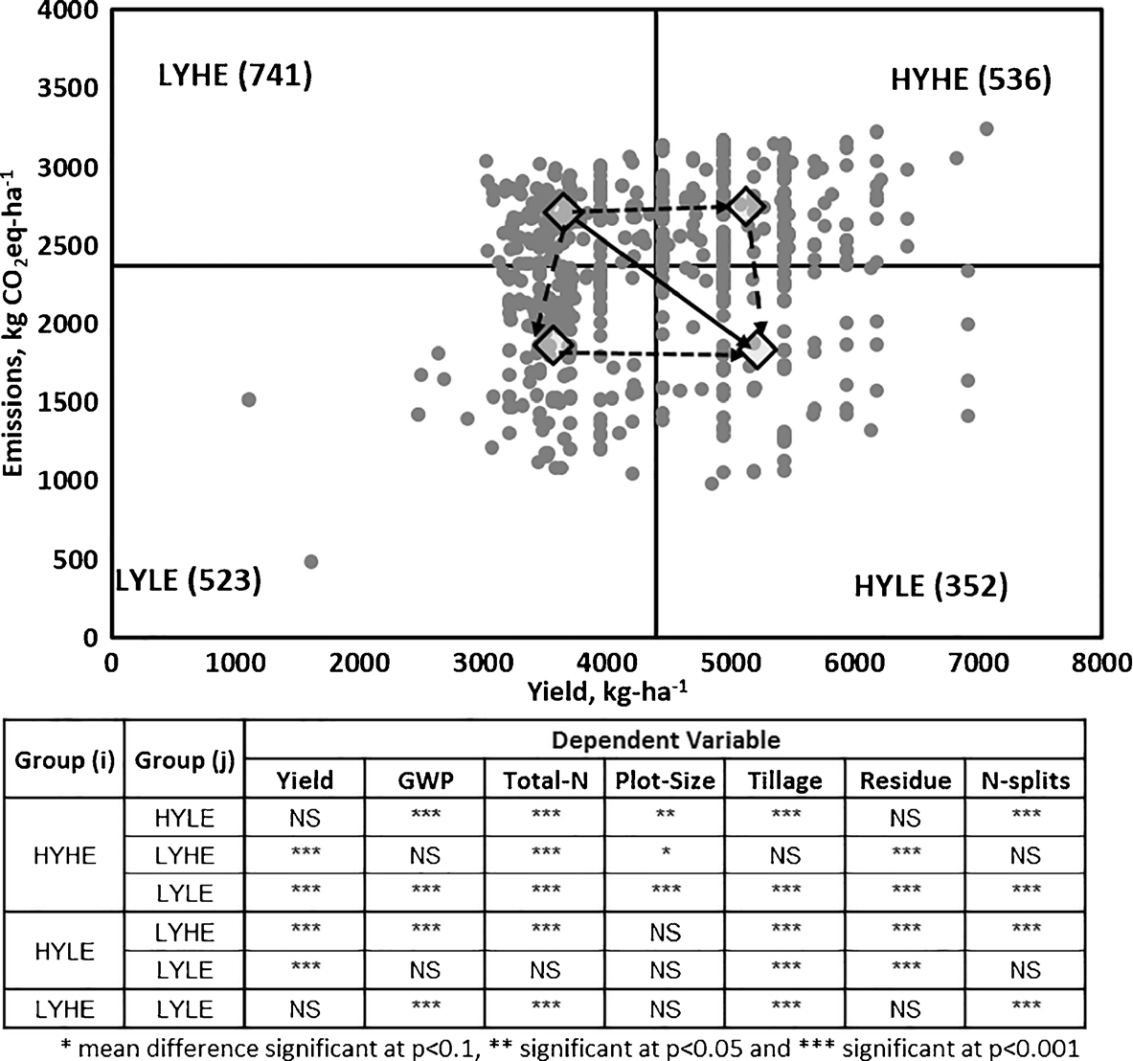
Grain yield-GHG emission framework demonstrating high-yield low-emission pathways. The *gray dots* represent grain yield and corresponding GHG emission for 630 rice farmers from Karnal. LYLE, LYHE, HYHE and HYLE represent low-yield low-emission, low-yield high-emission, high-yield high-emission and high-yield low-emission quadrants, respectively, determined based on average yield and emission of the area. The values in parenthesis are yield-scaled GWP (kg CO_2_e Mg^−1^). *Short-dashed*, *long-dashed* and *solid arrow* indicate production improvement, emission reduction and transformative pathways, respectively. *, ** and *** indicate that mean difference is significant at *p* < 0.1, *p* < 0.05 and *p* < 0.01, respectively. *NS* non-significant

To develop the pathways for emission-efficient production systems and identify the role of different variables for increasing production and reducing emissions, we present a ‘yield-GHG emission’ framework using rice production data from the high-input production system, i.e. Karnal, as an example. Taking the mean grain yield and GHG emissions, all rice farmers in Karnal were divided into four groups: low-yield low-emission (LYLE), low-yield high-emission (LYHE), high-yield high-emission (HYHE) and high-yield low-emission (HYLE) (Fig. 5). Yield-scaled GWP was the lowest in HYLE and the highest in LYHE, with LYLE and HYHE having intermediate yield-scaled GWP. Theoretically, developing high-yield low-emission production systems would require a shift towards HYLE, i.e. lower right quadrant of Fig. 5. For this, the farmers in HYHE quadrant can follow an emission reduction (without compromising yield) pathway (long-dashed arrow in Fig. 5) whereas those in LYLE quadrant can follow yield improvement (with no additional emission) pathway (short-dashed arrow in Fig. 5). The farmers in LYHE quadrant, on the other hand, can follow production improvement pathway, emission reduction pathway or even transformative pathway (increase production and reduce emission) (solid arrow in Fig. 5) depending on production condition and resources available. Zero-tillage (ZT) is primarily responsible for low emissions, both in low and higher yield regimes. None of the farmers adopting ZT fell into the high emission quadrants (i.e. LYHE and HYHE) indicating that adoption of ZT is one of the ways towards low-emission pathway. As stated earlier, the higher emissions with higher number of N splits were mainly due to the confounding of number of N splits and total amount applied with farmers using more N splits also using a greater amount of N. Farmers in HYHE and HYLE quadrants retained more crop residues supporting the view that residue retention is an essential component of sustainable intensification in tropical agro-ecosystems (Powlson et al. 2016). Larger plot sizes in general resulted in lower emissions indicating higher input use efficiencies confirming the findings of Ju et al. (2016) from China.

As cereal seeds contain large amount of storage protein reserves and protein comprises about 6% N (Ladha et al. 2016), more production will require more N uptake. However, significantly higher rate of N application by the farmers in LYHE quadrant than those in HYHE quadrant clearly demonstrates that higher total N does not necessarily result in higher yield but does lead to higher emissions. This is because crop yield is likely to be limited by other factors including other nutrients, water and soil conditions. Precision N use (right source, right time and right method of application) could be a transformative pathway for farmers in LYHE quadrant to increase production and reduce GHG emission. Our results in Fig. 3 show that N application between 80 and 100 kg N per ha (both in Karnal and Vaishali) provides the highest grain yield with the lowest GHG emission intensity in rice and between 120 and 140 kg N per ha in Karnal and 80–100 kg N per ha in Vaishali for the same in wheat, indicating the optimum N rate for yield optimization and emission reduction. However, the majority of the farmers in the study area (95 and 82% farmers growing rice and wheat, respectively, in Karnal and 23 and 40% farmers growing rice and wheat, respectively, in Vaishali) reported application rates that exceed the optimum rate of N in rice and wheat. Part of the additional N fertilizer required to increase N uptake in order to increase production can be offset to some extent by management practices that improve N use efficiency (Mueller et al. 2014). In this line, our results show huge opportunities to reduce GHG emission whilst maintaining grain yield by reducing N rate and adopting best fertilizer management practices to increase nutrient-use efficiency.

## Policy implications

In the context of climate change, agriculture and food security, our findings have several important policy implications in relation to the following:

(i) farmers’ costs of production and increasing risk of future climate change

Cost of fertilizer is increasing over time and so supra-optimal application of fertilizer represents a wasted cost of production. At the level of an individual farmer, it may not be significant, but collectively, it is a huge cost. As seen in Haryana where 82 to 95% of the farmers were applying above the optimal rate, this leads to a huge loss of agricultural inputs and farm household income in addition to all the possible negative environmental externalities (Sapkota et al. 2014). Moreover, it has implications for climate change, which ultimately affects farm production and increases the burden of adaptation in the future.

(ii) the government in terms of integrating policies and technology and enhancing farmers access to new technology and information

Many developing countries’ Intended Nationally Determined Contributions (INDCs) to the UNFCCC’s Paris Agreement have identified agriculture and allied sectors as one of the priority areas for emission reduction. Our case study from low- and high-input production systems indicates that if commercialization of agriculture in low-input agriculture follows a similar pathway to that of high-input production where rates of fertilizer use are supra-optimal, then targets for reductions in GHG emissions will be even more difficult to achieve. Therefore, the governments need to set up alternative pathways for agricultural development so that high-yield, low-emission targets are achieved in the agricultural sector. For example, optimum fertilizer application in wheat using optical sensors such as the GreenSeeker can reduce national emissions in India by 0.14 to 2.5 million Mg CO_2_e without compromising yield (Basak 2016). However, such alternative pathways should not only focus on technology but also on the socio-economic and human behavioral dimensions. Our results show strong associations between grain yield and emissions with various socio-economic and household characteristics such as education and gender and access to information (mainly ICT).

(iii) agricultural research community and civil society

Coupled with the immediate task of tackling widespread poverty and nutritional insecurity, there is the pressing challenge of increasing farmer awareness of new technologies in developing countries. Governments need to work together with the international community to disseminate appropriate technologies that help farmers take rational decisions to make agricultural production sustainable. Our findings add to the understanding of the social drivers contributing to climate change, particularly in relation to GHG emissions from agriculture. We found that education and access to information are important factors affecting crop yield and emissions. There is a need to educate farmers about the adverse impact of adopting inappropriate technologies. Farm cooperatives and local non-governmental organizations can play a vital role here. The Governments need to support local organizations to carry out on-farm training courses. ICT can be used to inform farmers on how they can make savings in terms of reduced cost of input (e.g. fertilizer) and increase income through higher yields.

## Conclusion

The global target for reducing agricultural emissions to limit global warming in 2100 to 2 °C above pre-industrial levels is ca. 1 gigatonne of CO_2_ equivalent per year by 2030 (Wollenberg et al. 2016). Low emission development (LED) in agriculture with the adoption of appropriate technologies and practices can deliver a large portion of the needed mitigation. For this, agricultural production systems must consider the impacts of adaptive interventions not only in terms of the primary goal of food security but also in terms of GHG emissions. This study explores the options that can help to reduce agricultural emissions whilst raising production and in a region of global importance in relation to the food-climate nexus. Our study highlights the contribution that improved cropland management can make to India’s INDC of emissions reduction to UNFCCC.

Growth in food demand and agricultural emissions is projected to be among the highest in Asia and most particularly in the Indian subcontinent. High-yield low-emission pathways are required to address the rapidly increasing demand for food and global climate change. Our analyses clearly indicate that in the case of agriculture, a high-yield low-emission pathway is possible through wide-scale adoption of improved technologies and practices such as tillage, irrigation, residue, farm manure and nitrogen fertilizer application and management.

All mitigation-related interventions require investment decisions at the household level. Our analyses show that the implementation of emission-reducing technologies and practices are influenced by the household’s socio-economic conditions including family size, gender of household head and farm size as well as flow of information through training and use of ICT. These socio-economic factors must be taken into account when considering the scaling out of mitigation-related interventions and the implementation of high-yield low-emission pathways in agriculture. Future research evaluating a high-yield low-emission pathway in agriculture should consider not only emission-reducing interventions but also the tradeoffs between GHG emissions and food/nutrition security in different agricultural production systems.

## Electronic supplementary material

Figure S1 (PNG 995 kb)
