# Supplementary figures and images for: Identifying high-yield low-emission pathways for the cereal production in South Asia
Source: Mitig Adapt Strateg Glob Chang. 2017 Jul 22;23(4):621–41. doi: 10.1007/s11027-017-9752-1 (PMC6054015; doi:10.1007/s11027-017-9752-1)

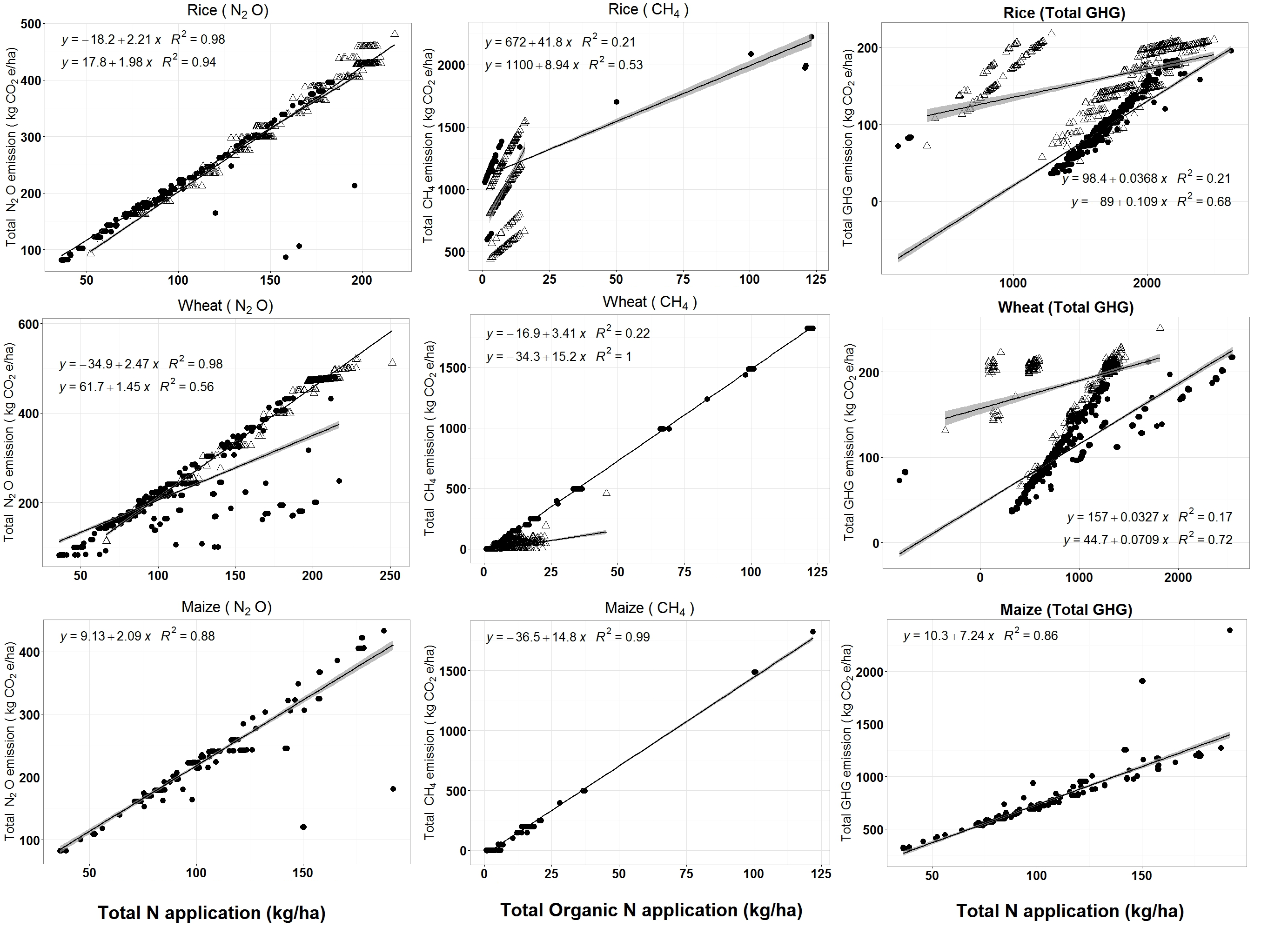

Supplement: Supplementary file 1 — (PNG 995 kb) [file 11027_2017_9752_MOESM1_ESM.png]
